# Gene Expression Clustering and Selected Head and Neck Cancer Gene Signatures Highlight Risk Probability Differences in Oral Premalignant Lesions

**DOI:** 10.3390/cells9081828

**Published:** 2020-08-03

**Authors:** Andrea Carenzo, Mara S. Serafini, Elisa Roca, Alberto Paderno, Davide Mattavelli, Chiara Romani, Pierre Saintigny, Senada Koljenović, Lisa Licitra, Loris De Cecco, Paolo Bossi

**Affiliations:** 1 Integrated Biology Platform, Department of Applied Research and Technology Development, Fondazione IRCCS Istituto Nazionale dei Tumori, 20133 Milan, Italy; andrea.carenzo@istitutotumori.mi.it (A.C.); Mara.Serafini@istitutotumori.mi.it (M.S.S.); 2 Department of Medical & Surgical Specialties, Radiological Sciences & Public Health, University of Brescia, ASST Spedali Civili, 25123 Brescia, Italy; elisaroca@gmail.com (E.R.); paolo.bossi@unibs.it (P.B.); 3 Unit of Otorhinolaryngology-Head and Neck Surgery, Azienda Socio Sanitaria Territoriale (ASST), Spedali Civili di Brescia, Department of Medical and Surgical Specialties, Radiologic Sciences, and Public Health, University of Brescia, 25123 Brescia, Italy; albpaderno@gmail.com (A.P.); davide.mattavelli@unibs.it (D.M.); 4 Angelo Nocivelli Institute of Molecular Medicine, University of Brescia and ASST-Spedali Civili of Brescia, 25123 Brescia, Italy; cromani76@gmail.com; 5 Centre Léon Bérard, Centre de Recherche en Cancérologie de Lyon, University Lyon, Université Claude Bernard Lyon 1, INSERM 1052, CNRS 5286, 69100 Lyon, France; Pierre.SAINTIGNY@lyon.unicancer.fr; 6 France & Department of Medical Oncology, Centre Léon Bérard, 69100 Lyon, France; 7 Department of Pathology, Erasmus University Medical Center, University Medical Center Rotterdam, 3000 Rotterdam, The Netherlands; s.koljenovic@erasmusmc.nl; 8 Head and Neck Cancer Medical Oncology 3 Department, Fondazione IRCCS Istituto Nazionale dei Tumori (INT), 20122 Milan, Italy; lisa.licitra@istitutotumori.mi.it; 9 Department of Hematology and Oncology, University of Milan, 20122 Milan, Italy

**Keywords:** biomarkers, oral preneoplastic lesion, gene expression profiling, head and neck squamous cell carcinoma

## Abstract

Background: Oral premalignant lesions (OPLs) represent the most common oral precancerous conditions. One of the major challenges in this field is the identification of OPLs at higher risk for oral squamous cell cancer (OSCC) development, by discovering molecular pathways deregulated in the early steps of malignant transformation. Analysis of deregulated levels of single genes and pathways has been successfully applied to head and neck squamous cell cancers (HNSCC) and OSCC with prognostic/predictive implications. Exploiting the availability of gene expression profile and clinical follow-up information of a well-characterized cohort of OPL patients, we aim to dissect tissue OPL gene expression to identify molecular clusters/signatures associated with oral cancer free survival (OCFS). Materials and methods: The gene expression data of 86 OPL patients were challenged with: an HNSCC specific 6 molecular subtypes model (Immune related: HPV related, Defense Response and Immunoreactive; Mesenchymal, Hypoxia and Classical); one OSCC-specific signature (13 genes); two metabolism-related signatures (3 genes and signatures raised from 6 metabolic pathways associated with prognosis in HNSCC and OSCC, respectively); a hypoxia gene signature. The molecular stratification and high versus low expression of the signatures were correlated with OCFS by Kaplan–Meier analyses. The association of gene expression profiles among the tested biological models and clinical covariates was tested through variance partition analysis. Results: Patients with Mesenchymal, Hypoxia and Classical clusters showed an higher risk of malignant transformation in comparison with immune-related ones (log-rank test, *p* = 0.0052) and they expressed four enriched hallmarks: “TGF beta signaling” “angiogenesis”, “unfolded protein response”, “apical junction”. Overall, 54 cases entered in the immune related clusters, while the remaining 32 cases belonged to the other clusters. No other signatures showed association with OCFS. Our variance partition analysis proved that clinical and molecular features are able to explain only 21% of gene expression data variability, while the remaining 79% refers to residuals independent of known parameters. Conclusions: Applying the existing signatures derived from HNSCC to OPL, we identified only a protective effect for immune-related signatures. Other gene expression profiles derived from overt cancers were not able to identify the risk of malignant transformation, possibly because they are linked to later stages of cancer progression. The availability of a new well-characterized set of OPL patients and further research is needed to improve the identification of adequate prognosticators in OPLs.

## 1. Introduction

Many cancers evolve from their precursor lesions and have a natural history of progression that provides a window of opportunity for intervention [1]. The biological mechanisms underlying this evolutionary trajectory can only be clearly understood through extensive characterization of the molecular, cellular, and non-cellular properties of pre-cancer and cancer, including the contribution of the microenvironment (stromal cells, immune cells, etc.) [2].

Oral premalignant lesions (OPL), currently defined as oral potentially malignant disorders, represent the most common oral precancerous conditions [3]. OPL have been clinically described as oral leukoplakias and erithroplakias, consisting of lesions that present as white or red patches, having excluded other known diseases or disorders that carry no increased risk for cancer [4].

The OPL malignant transformation potential is highly variable according to different studies, highlighting the difficulty in defining lesions at higher risk and in providing strict clinical and/or molecular prognostic factors [5,6,7]. However, a recent systematic review estimated the overall mean proportion of malignant transformation rate for oral leukoplakia as 9.7% (7.8–11.7%) [8]. Unfortunately, the severity of the histologic alterations of these lesions in general, and in the oral cavity in particular, is poorly reproducible, resulting in its limited value as a predictive factor for malignant transformation [9].

In contrast to overt head and neck squamous cell carcinoma (HNSCC), very few studies have been performed to comprehensively profile the molecular and cellular alterations in premalignant lesions. Among them, somatic mutations as well as chromosomal copy number aberrations have been identified, with loss of heterozygosity (LOH) being the most effective in predicting progression to oral cancer [10].

Furthermore, alterations in gene expression of OPL have been shown early during oral tumorigenesis and have been associated with malignant transformation, leading to possible gene expression profiles predicting oral cancer-free survival (OCFS). This predictive model was shown to improve the clinicopathologic determinants of oral malignant transformation [11].

To the best of our knowledge, in OPLs only four datasets of gene expression are at present publicly available and only one of them includes a significant sample size with complete clinical annotations [12,13,14,15]. In the latter, case material was deeply analysed at molecular level in a subsequent paper [16]. The two identified subtypes, immunological and classical, did not show predictive value in terms of OCFS. However, decreased miRNA-142-5p expression and lower T-cell, monocytic and myeloid dendritic cell immune infiltration associate with OCFS within the immunological subtype, while LOH at 3p14, LOH at 17p13 and TP53 are prognosticators for OCFS within the classical subtype.

We identified six molecular subtypes in HNSCC based on a meta-analysis of HNSCC gene expression data and after a decomposition process to infer the disease component (i.e. distance from the normal state) from each tumor sample [17]. The six molecular subtypes differed in terms of de-regulated genes, enriched biological pathways and overall survival and were defined as Cl1 (HPV-like), Cl2 (Mesenchymal), Cl3 (Hypoxia), Cl4 (Defense Response), Cl5 (Classical/Smoking), and Cl6 (Immunoreactive). The best overall survival was observed in HPV-like, and the worst in Cl2 and Cl3. When progression analysis of disease (PAD) [18], a tool to disclose topological connections in high dimensional data, was applied and the distance between the six clusters from normal tissue was calculated, HNSCC tumors associated with a linear progression, displaying tumors close to the normal state (Cl6) and ending with tumors with the largest deviation from the normal state (Cl2). This analysis suggested the possibility to discern different stages, modulated by gene expression, during HNSCC progression, with a consequent gain in aggressiveness [19].

Thus, exploiting the availability of gene expression profiles and clinical information in a well-characterized set of OPL patients, we aimed at dissecting tissue OPL gene expression to identify molecular clusters/signatures associated with OCFS [15]. To approach the understanding of the relationship between biological mechanisms behind the disease progression, from premalignant lesion to oral cancer, we tested the six HNSCC molecular subtype models along with recent signatures related to prediction of overall survival in oral cancer patients. 

## 2. Materials and Methods

### 2.1. OPL Dataset

Eighty-six oral premalignant cases, defined as OPL and enrolled in a randomized chemoprevention trial at University of Texas MD Anderson Cancer Center (Houston, TX 77030, USA), were profiled by microarray gene expression [20]. Lesions included in the trial had: clinical and histologic evidence of measurable or assessable OPLs (leukoplakia and/or erythroplakia); histologic examination showing dysplasia or extensive leukoplakia with hyperplasia and symptoms (i.e., pain), cosmetic cases (e.g., leukoplakia of the lips), or high-risk location (i.e., soft palate, floor of mouth, ventral tongue, and alveolar ridge). A normalized data matrix was retrieved from NCBI Gene Expression Omnibus repository (ID: GSE26549), where raw data were processed by quantile normalization and RMA algorithm; then, expression values were log_2_ transformed [21,22].

### 2.2. Gene Expression-Based Molecular Subtyping

In a previous study on a meta-analysis of 8 datasets including 527 HNSCC tumors and 138 normal tissues, we used a data structure decomposition approach using the Disease-Specific Genomic Analysis (DSGA) tool [23]. Gene expression profiling depicts the average transcriptomic landscape weighted by the cell type proportions present into bulk tissue specimens [24]; DSGA method allows simplifying the expression data in order to highlight the pathologic component embedded into the data by isolating the disease-like and the normal-like portions for every gene. Taking into account the expression data of normal tissues, DSGA builds a healthy state model that allows each tumor tissue to be decomposed as the sum of two components: (i) the normal component; (ii) the disease component (i.e., residuals). As a result, the disease component assessed the extent to which each tumor deviates from the normal state and was used for the identification of our six molecular subtypes. Subsequently, we defined the topological connections among HNSCC samples, in order to study the structures into the high-dimensional data, recognizing shapes and patterns leading, eventually, to the identification of meaningful subgroups. To this purpose, we applied PAD, a topological approach that discloses the geometry into the data and provides an easily accessible picture of data relationships [18]. PAD incorporates Mapper, a mathematical tool that identifies local clusters and then defines the interaction between these clusters by connecting them to form a graph whose shape captures the topology present into the data [25]. Through PAD, a subset of genes with significant deviation from the healthy state was retained and used to disclose the topological connections in our HNSCC meta-analysis. Six different HNSCC subtypes that summarize the aberrant alterations occurring during tumor progression were identified. Based on their main biological characteristics and de-regulated signaling pathways, the subtypes were designed as: (i) immunoreactive; (ii) defense response; (iii) HPV-like; (iv) classical (smoking related); (v) hypoxia; (vi) mesenchymal. Our findings show that HNSCC tumors are topologically associated through a linear progression starting from tumors having molecular features close to the normal state and ending with those with large deviation from the normal state, suggesting an increase in alterations accumulated during different stages of tumor progression. The distance from the normal state is summarized by the PAD score. Since our subtyping model in HNSCC evaluates the deviation of each tumor from the continuous range of normal phenotypes, its application on OPLs with oral cancer development as clinical endpoint is conceivable. 

Based on the molecular subtyping model developed in HNSCC, the topological distances (i.e., PAD score) in the GSE26549 cases were predicted by least-angle regression (LARS), an algorithm for fitting linear regression models to high-dimensional data (i.e., gene expression). LARS is useful in predicting quantitative traits: it avoids the over-fitting issues of least-squares linear regression when the number of variables (i.e., genes) is large compared to the number of cases, introducing cross-validated squared predictions [26]. LARS was applied using a dedicated BrB-plugin that develops a linear model for predicting a continuous response variable based on gene expression measurements, imposing 0% as error threshold and 10-fold cross-validation for model selection [27].

As described in [17], Prediction Analysis for Microarrays (PAM) algorithm [28] was applied to project our classification to GSE26549. PAM, a nearest shrunken centroid method, is a popular classification method for high-dimensional data. The approach relies on a classification rule based on the scaled distance between the expression profiles of new samples and class centroids. In deeper detail, PAM shrinks the class centroids towards the overall means and embeds a variable selection mechanism [29]. The degree of shrinkage allows minimizing the cross-validated error rate on the training set [30]. The association between PAD score and subtype membership or other clinical/pathological features available on GSE26549 was assessed by Kruskal–Wallis test. 

### 2.3. Gene Set Enrichment Analysis (GSEA) Functional Analysis

To disclose the de-regulated biological pathways in the highest risk subtypes compared to the lowest risk subtypes, we performed a functional characterization of the Hallmark gene set collection using gene set enrichment analysis (GSEA) [31,32]. We considered as significantly enriched those gene sets having a normalized enrichment score (NES) > |1.5|, and a False Discovery Rate (FDR) < 0.25 (25%). To run the analyses, we used GSEA version 4.0.3 with 10,000 phenotype permutations and “tTest” as metric for ranking genes.

### 2.4. Tumor Microenvironment Analysis

Immune and stroma cell abundance in OPLs was estimated through the xCell R package [33]. xCell is based on single-sample GSEA to estimate the abundance of 64 immune cell types, including adaptive and innate immune cells, hematopoietic progenitors, epithelial cells, and extracellular matrix cells using a novel compendium of 489 gene sets. We focused on 26 cell types provided by the xCell tool corresponding to the “parent” cell types and on three summary scores (immune, stroma and microenvironment score).

### 2.5. Prognostic Signatures 

A number of signatures on oral cancer were developed based on biological knowledge and providing evidence on their prognostic value. After a survey of the literature, we investigated the potential prognostic association of the following signatures: (i) a model including 13-gene signature and implemented with age and gender for HPV-negative early stage OSCC [34]; (ii) metabolism-based prognostic risk score (MPRS) based on six metabolic pathways (hsa00290: Valine, leucine and isoleucine biosynthesis, hsa01230: Biosynthesis of amino acids, hsa00430: Taurine and hypotaurine metabolism, hsa00380: Tryptophan metabolism, hsa00232: Caffeine metabolism, hsa00534: Glycosaminoglycan biosynthesis—heparan sulfate/heparin) [35]; (iii) hypoxia metagene containing 99 genes [36]; (iv) lipid metabolism-related signature [37]. Further details can be found in Table S1. The genes were mapped using EntrezID annotation [38].

### 2.6. Variance Partition Analysis

There are likely many variables that contribute to variability in OPL between individuals. To assess the contribution of the prognostic molecular signatures and clinical features as a source of data variance, we adopted a visualization framework. Thus, the variancePartition package was used in order to evaluate the proportion of gene expression variance explained by both the demographic/clinical (i.e., age, gender, smoking, alcohol, histology) and the molecular covariates [39]. The variancePartition package uses linear mixed model based statistical methods to quantify the contribution of multiple sources of variation. This method is instrumental in providing a way for comparing the relative effects of molecular and demographic/clinical drivers on gene expression.

### 2.7. Bioinformatics Analysis and Data Visualization

Bioinformatics analysis was performed using R version 3.6.3 [40], and BrB-ArrayTool version 4.6.0, developed by Dr. Richard Simon and the BRB-ArrayTools Development Team, available at http://linus.nci.nih.gov/BRB-ArrayTools.html [41].

The R/Bioconductor package ComplexHeatmap [42,43] was used to visualize data and to assess whether there was association between the expression values of 102 genes in the 86 OPL samples and clinical-molecular features, including molecular subtype membership, gender, histology, and smoking and alcohol habits. Genes (rows) were chosen by PAD analysis and clustered by Pearson’s correlation distance, while samples (columns) were ordered by increasing PAD score. 

Regarding the published signatures, the sum of the expression values weighted by the regression coefficients of each gene entering into the signatures was assessed to compute the risk scores for 13-gene model and MPRS. When the algorithm was not specified (i.e., hypoxia metagene and lipid metabolism-related signature), the samples were ranked by the z-score based on the genes. The GSVA R package was used to estimate the z-scores of a gene set over the samples in an unsupervised manner [44].

For biomarker cutoff determination, we used the Cutoff Finder R package (available at http://molpath.charite.de/cutoff) that allows optimizing a cutoff point taking into account the presence of complex distributions in the data using the time interval for oral cancer development as endpoint [45].

Kaplan–Meier survival analyses for both the six molecular subtypes and the four prognostic signatures were realized through the R package survival [46]. The ggplot2 package [47], was used to represent the OCFS curves and a follow-up of 8 years was chosen as a limit because of the very low number of events occurring after that time period.

## 3. Results

### 3.1. Six Molecular Subtypes 

**Figure 1:**
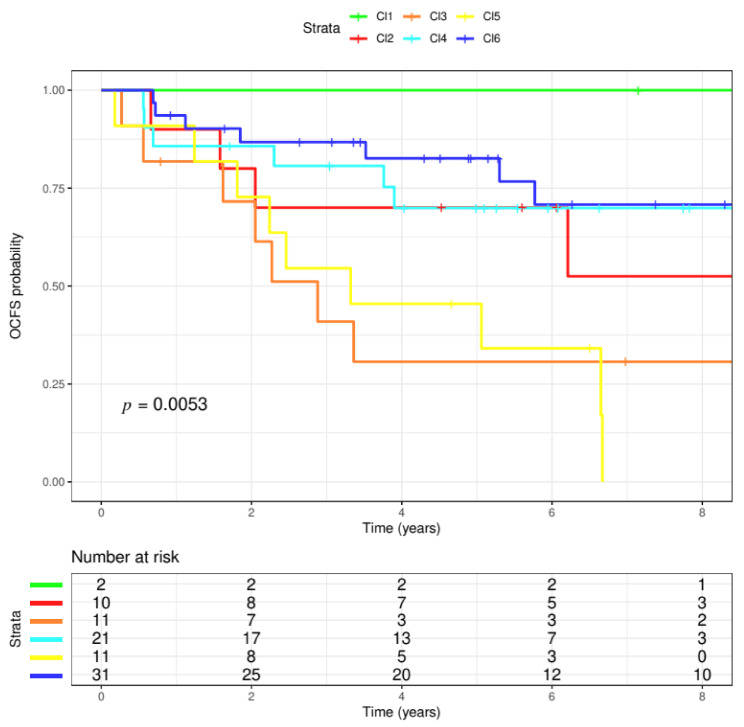
Six molecular subtypes. The six molecular HNSCC (head and neck squamous cell cancers) subtype classifications were applied to the OPL (oral premalignant lesions) dataset. The dataset included 86 patients, having a median follow-up of 6.08 years. The six clusters evidenced differences in terms of probability of oral cancer development (*p* = 0.0053, log-rank test). In particular, three clusters appeared to have the highest probability to modify their OPL status to cancer (Cl2, red; Cl3, orange; Cl5, yellow). On the contrary, the lowest probability was observed in Cl1, Cl4, Cl6 (green, blue, light blue, respectively).

The OPL dataset disposed of 86 patients’ gene expression data including histologically proven hyperplasia (63%) and dysplasia (37%) cases; among the latter, 21 were mild, 8 moderate and 2 severe. The median follow-up time was 6.08 years and 35 (40.7%) patients developed OSCC over the course. Cases were stratified according to a classification based on the six molecular subtypes [17]. As a result, 2 cases (2.3%) were classified as Cl1-HPV, 10 (11.6%) as Cl2-Mesenchymal, 11 (12.8%) as Cl3-Hypoxia, 21 (24.4%) as Cl4-Defense Response, 11 (12.8%) as Cl5-Classical, 31 (36.0%) as Cl6-Immunoreactive. The six subtypes applied to the OPL dataset evidenced differences in terms of OCFS probability (log-rank test; *p* = 0.0053). In detail, clusters that in the context of HNSCC appeared to have a worst survival (Cl2, Mesenchymal; Cl3, Hypoxia; Cl5, Classical), displayed the same pattern with a 5-year OCFS of 70% (95% CI: 46.7–100), 30.7% (95% CI: 12–78.3) and 45.5% (95% CI: 23.8–86.8), respectively. Conversely, a higher 5-year OCFS probability of 100%, 69.9% (95% CI: 52.3–93.4) and 82.6% (95% CI: 69.7–97.8) was observed in Cl1, Cl4 and Cl6 (HPV-like, Defense Response, Immunoreactive), respectively (Figure 1). Considering the small sample size of the dataset, we decided to perform the subsequent analyses comparing subtypes with low OCFS (high risk: Cl2, Cl3, Cl5) against subtypes with high OCFS (low risk: Cl1, Cl4, Cl6). 

### 3.2. Progression Analysis of Disease

**Figure 2:**
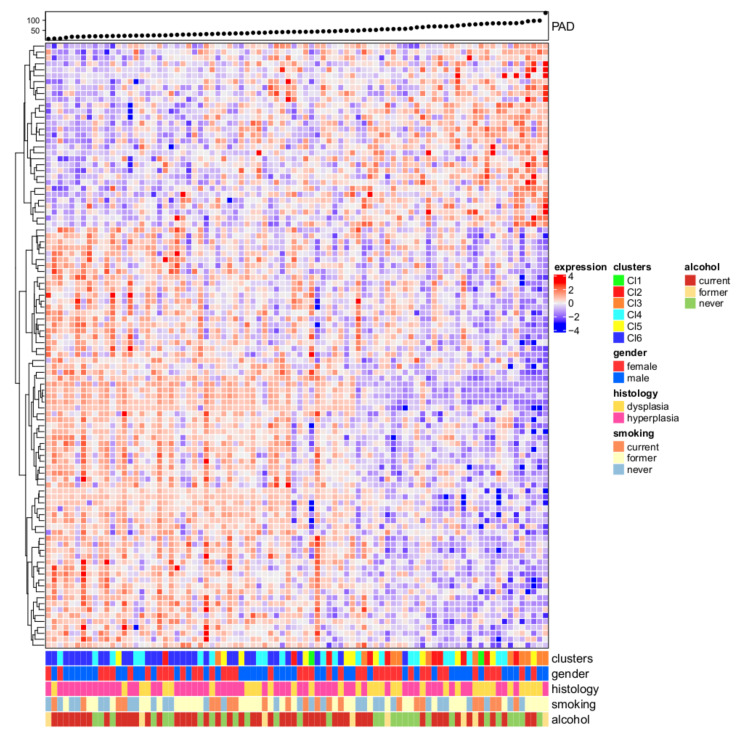
Progression analysis of disease (PAD). The upper panel shows the PAD score and the samples were ranked based on the increasing score. Low score values correspond to expression profiles close to the normal state; on the opposite, as the score values increase, the molecular differences compared to the normal state increase. The heatmap depicts the normalized gene expression values of 102 genes (rows) associated with the PAD procedure for the 86 OPL samples (columns) in a color scale ranging from blue to red, corresponding to low and high expression, respectively. On the bottom panel, five features were reported in a bar plot to highlight their association with increasing scores (i.e., cluster membership, gender, histology, smoking, and alcohol habits).

To unravel the topological characteristics hidden in the OPL gene expression data and to identify the relevant connections among them we associated the oral premalignant cases to PAD linear progression. Expression profiles belonging to the OPL dataset showed a linear progression starting from molecular features close to the normal state and ending with samples distant from the normal state, thus suggesting an accumulation in alterations through different stages of tumor progression. We imputed the topological distances from normal state highlighting 102 genes and Figure 2 shows the heatmap of the genes associated with PAD. OPL cases were ranked based on the topological transformation disclosing a significant association with subtype membership (*p* = 6.6 × 10^7^) with Cl6-Immunoreactive and Cl4-Defense response having profiles close to the normal state, and Cl3-Hypoxia being the most distant. Among the available clinical parameters (i.e., age, gender, smoking, alcohol habits, histology), PAD associates with histology (*p* = 0.0216) with dysplasia more distant to normal state compared to hyperplasia. 

### 3.3. Functional Analysis and Tumor Microenvironment Composition

To disclose the de-regulated biological pathways in the highest risk subtypes compared to the lowest risk subtypes, we performed a functional characterization using GSEA. The higher risk subtypes (Cl2, Cl3, Cl5) appeared to have four enriched Hallmarks: “TGF beta signaling” (NES = 1.72; FDR = 0.19), “angiogenesis” (NES = 1.64; FDR = 0.18), “unfolded protein response” (NES = 1.56; FDR = 0.24), “apical junction” (NES = 1.56; FDR = 0.19) (Figure S1). No pathways resulted enriched in the lower risk subtypes applying the same thresholds. Additionally, we applied xCell in order to evaluate if any difference in the tumor microenvironment composition was present in the six molecular subtypes, clustered in higher risk (Cl2, Cl3, Cl5) and lower risk (Cl1, Cl4, Cl6) groups. No differences were found when the three overall summary scores (i.e., microenvironment, immune, and stroma scores; Figure S2A) were considered. However, when we investigated the specific stromal/other cells and immune cellular components in detail, we found a significant difference between the high and low risk groups. Stromal components/other cells (Figure S2B) including skeletal muscle cells and mesenchymal stem cells (MSC), as well as melanocytes, are significantly differently abundant (*p* = 0.026, *p* = 0.0095, *p* = 9.6×10^−6^, respectively). Melanocytes are enriched in the low risk group, while skeletal muscle cells and MSC are in the high risk group. Regarding the immune component, monocytes resulted enriched in high risk group (*p* = 0.036) (Figure S2C). 

### 3.4. Prognostic Value of Oral Cavity Signatures 

**Figure 3:**
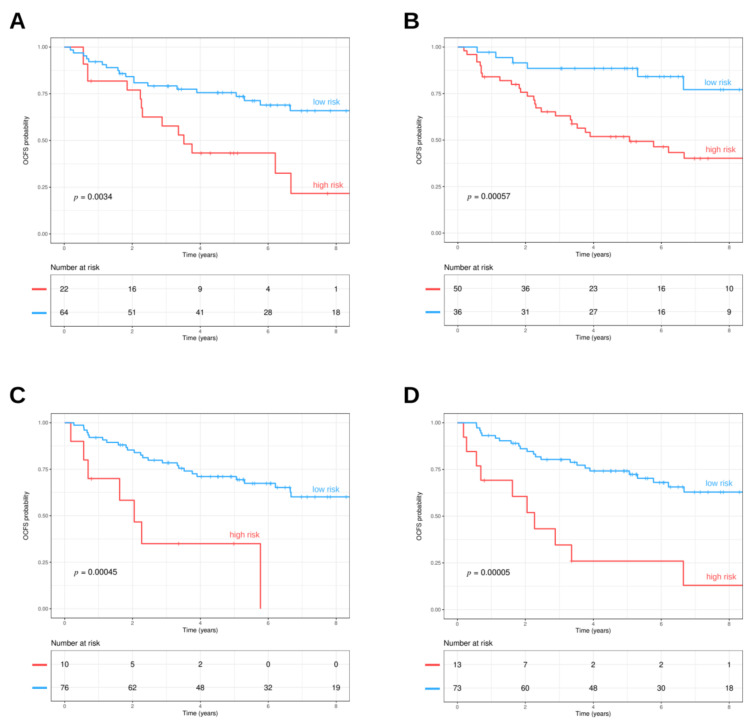
(**A**) 13-gene oral cancer signature. The prognostic model was applied to the OPL dataset and stratified patients in high (red line) and low (light blue line) risk groups (*p* = 0.0034, log-rank test). (**B**) Signature related to genes related to lipid metabolism. The three gene signatures (TGFB1, SPP1, SERPINE1) identified by Hu et al. were applied to the OPL dataset. The signature visualized two different trends in patients, those with lower (light blue line) and higher risk (red line) (*p* = 0.00057, log-rank test). (**C)** MPRS. The metabolic pathway signature (MPRS) based on 6 gene sets was applied to the OPL dataset. The MPRS stratified patients with lower (light blue line) and higher risk (red line) (*p* = 0.00045, log-rank test). (**D)** Hypoxia signature. The hypoxia signatures based on 99 genes were applied to the OPL dataset. The hypoxia signature stratified patients with lower (light blue line) and higher risk (red line) (*p* < 0.0001, log-rank test).

The 13-gene model originally developed in Lohavanichbutr et al. [48] was revised in Chen et al. [34] focusing on AJCC stage I/II cases, with the inclusion of clinical features (i.e., age and gender) along with the 13-gene signature: the final model resulted in improvement in the prediction of 2-year oral cavity cancer-specific survival. The Chen’s stage I/II model was tested in the OPL dataset. The model was structured for predicting the survival of HPV-negative OSCC patients; however, it proved to perform well also in the context of OPL setting (Figure 3A). The 13-gene model significantly stratified patients with higher and lower risk (HR = 2.71, 95%CI: 1.36–5.41, *p* = 0.0034).

Additionally, the prognostic molecular signatures related to metabolisms were applied. Hu et al. [37] reported a signature associated with obesity that included genes related to lipid metabolism. This signature assesses the expression of three genes (TGFB1, SPP1, SERPINE1) in OSCC. The model was tested in the GSE26549 dataset in order to understand its role in identifying the risk of oral cancer development. The three gene lipid metabolism-related signatures were able to identify two different risk trends, stratifying patients with lower or higher risk (HR = 4.18, 95%CI: 1.73–10.1, *p* = 0.00057) (Figure 3B). Moreover, we tested the six metabolic pathway signatures (MPRS) identified by Xing et al. [35] to foresee prognosis in HNSCC patients. The metabolism-related signature was able to stratify patients with lower and higher risk (HR = 4.16, 95%CI: 1.76–9.82, *p* = 0.00045) (Figure 3C). Finally, high and low risk groups were obtained by the 99 genes hypoxia signature and their survival distributions were compared, finding a statistically significant difference (HR = 4.22, 95%CI: 1.99–8.93, *p* = 0.00005) (Figure 3D).

### 3.5. Variance Partition Analysis 

**Figure 4:**
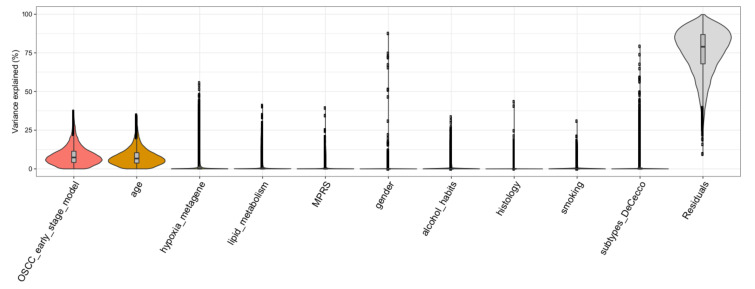
Violin plots depicting drivers of variation in gene expression. *X*-axis shows various traits and *y*-axis shows the percentage of variance explained. Thickness of each violin bar depicts the proportion of genes corresponding to level of *y*-axis.

In order to determine to which extent the gene expression is affected by clinical and biological factors including their relationship to molecular cluster architecture (i.e., gender, age, smoking, alcohol_habits, histology, subtypes_DeCecco, hypoxia_metagene, MPRS, lipid_metabolism, OSCC_early_stage_model), a variance partition analysis was carried out. Taking into account the whole gene expression data, the OSCC 13-gene model and age explained a substantial percentage of the variance in gene expression (7.37% and 6.68%, respectively); however, the residuals (percentage not explained by the variables under investigation) reached a median of 79% (Figure 4), showing that most of the gene expression remains to be explained and is independent to the investigated biological or clinical features.

## 4. Discussion

Oral leukoplakia and erythroplakia have a non-negligible prevalence in the general population: 2–3% and 0.02–0.83%, respectively [49]. In view of the significant diffusion of these alterations, even a low rate of progression to oral carcinoma may have a significant impact on global health. For this reason, risk stratification through comprehensive characterization of each lesion has the potential to significantly improve treatment and follow-up [50]. 

However, conventional characterization and diagnosis of OPLs is significantly hampered in two different levels. Firstly, clinical examination has limited value in detecting malignant potential of OPLs since their macroscopic appearance often does not reflect their histopathologic and molecular features; despite this, evaluation of OPLs is still largely based on simple mucosal inspection [51]. Secondly, histopathology is highly subjective and is not capable of providing a reliable measure of risk in a specific lesion [52]. 

Therefore, the definition of predictive factors of OPL malignant transformation is crucial both to identify high risk patients early and to reveal molecular pathways that may be regulated to prevent oral cancer development.

Concentrating the early diagnostic, therapeutic, and control efforts towards lesions that are potentially more dangerous is key in the strategies of cancer secondary prevention [53]. In this regard, OPLs with a particularly high-risk profile may be managed using the same principles as early stage oral cancer, preventing more extensive treatment, thus improving functional outcomes and prognosis.

On the other hand, OPLs with low risk of malignant progression could be just followed or managed in a less aggressive way, thus reducing inherent treatment morbidity and associated costs.

Molecular research in the setting of OPL is the necessary step to implement new preventative strategies [54]. 

We dissected the molecular pathways of a publicly available OPL dataset profiled by gene expression, and we investigated whether and to what extent the molecular portraits of HNSCC are already present in OPL. Our six HNSCC subtype stratification were identified after bioinformatics topological decomposition, transforming expression data of tumor tissue as a sum of the normal component that mimics healthy tissue, and the disease component that measures the deviation from normal state [17]. Considering the uniqueness of our approach in assessing the distance to normal state, we investigated the performance of our HNSCC subtype stratification in OPL. Having as endpoint OCFS and based on our subtypes stratification, we were able to identify three clusters at higher risk of oral cancer development, namely Hypoxia, Mesenchymal and Classical clusters, and the protective effect of immune-related clusters (i.e., Immunoreactive and Defense Response). A subset of 102 genes was disclosed as associated with the PAD score and, through PAD, the topological relationships among samples can be unveiled, reflecting the progression starting from normal state [18]. Our findings demonstrate that Immunoreactive and Defense response clusters share profiles close to the normal state, while the Hypoxia cluster is the most distant. PAD score associates with histology and cases having dysplasia show topological features distant from normal state compared to hyperplasia. When other available clinical parameters (i.e., age, gender, smoking, alcohol habits) were investigated, no association with PAD was found.

A number of signatures have been published providing evidence on their prognostic role in HNSCC [55]. Thus, we tested a number of biologically oriented signatures to verify their ability to stratify patients based on their risk of developing oral cancer. Our results highlight the role of hypoxia as a late event in OPL progression with high risk of cancerization that was confirmed by a 99-gene hypoxia signature [36]. Classical subtype is associated with smoking and metabolism-related pathways [17] and recently two studies reported prognostic value of metabolism pathways in HNSCC [35,37]. Our results show that both models provided a significant capability in stratifying high risk patients. In addition, we tested a model based on expression of 13 genes that was developed specifically for oral cancer prognosis [48]. The model has been recently revised to include demographic features (i.e., age and gender) providing improved performance in early stage tumors [34]. Considering its value in stage I/II stage oral cancer, we tested the 13-gene model in OPL, demonstrating its significant performance in defining high-risk oral premalignant lesions. Taken together, our results highlight the linear progression in OPL that can be disclosed by gene expression data and summarized with pathways or signatures developed in the tumor state. 

Although we identify gene expression based models associated with OCFS, the relevance of these models in accounting the variability of expression data has to be assessed. We exploited a tool implementing a statistical method to quantify the contribution of multiple sources of variation; clinical parameters (i.e., gender, age, smoking, alcohol habits, histology) and biological features (i.e., six molecular subtypes, hypoxia metagene, MPRS, lipid metabolism, 13-gene OSCC early stage model) were investigated as drivers of variation in gene expression data and they account for 21% of data variability, while 79% of variability is not explained by these covariates.

It is interesting to note the role of immune-related clusters as being protective against oral malignant transformation. OPL may be considered the equilibrium phase of the immunoediting concept, i.e., a dynamic process between the tumor cells and the immune system including, at the two opposites of the spectrum, the surveillance by immune system on one hand and tumor progression on the other [56]. Thus, an imbalance in the immunosuppressive microenvironment is the possible key explanation for malignant transformation. In this regard, the activation of the PD-1/PD-L1 pathway has a central role, witnessed by the expression of PD-L1 by multiple cell types within the microenvironment of OPL (tumor-associated macrophages, fibroblasts, lymphocytes) and by the fact that PD-L1 expression in epithelial and subepithelial cells is associated with malignant transformation [57]. 

Globally, an increased number of T-cells have been identified in OPL not transforming into cancer, in comparison with OPL, which showed malignant transformation, thus supporting the importance of immuno-mediated antitumoral mechanisms [58]. Moreover, the abundance of MSCs in the OPL at higher risk of malignant transformation justifies the shift towards a more immunosuppressive microenvironment, sustained by a process of epithelial-mesenchymal transition [59].

A comprehensive molecular knowledge of OPL will offer the possibility to explore new algorithms of interaction and mutual reinforcements among different therapeutic weapons such as surgical treatment, and systemic prevention by immunotherapy and targeted therapy. Until now, among molecular markers, the most effective in predicting oral cancer risk is LOH, which may appear in different histological grades of dysplasia. Patients carrying OPL with LOH at 3p14 and/or 9p21 plus LOH at another locus have an expected 3-year risk of developing oral cancer of 35% [60,61]. However, no direct implication may be derived from the discovery of high-risk OPL, as neither a different follow-up nor any systemic therapies are available.

We acknowledge the limitations of our study, mainly consisting of the analysis of a limited series of OPLs in which variance partition analysis proved that clinical, pathological, and molecular features of OPLs are able to explain only a limited portion of data variability. As OPLs are easily accessible lesions for clinical visits and monitoring, the concept of chemoprevention has been an attractive topic [62]. However, at present, the literature data are very scant in this field and the analyzed series is the only one having recruited prospective cases within the frame of a clinical study with strict inclusion criteria, with available well-annotated clinical data and with an appropriate follow-up. In addition, as the process of carcinogenesis can require a long period of time and can be quite unpredictable, our data seem to suggest an extension of OPL follow-up.

In conclusion, we strongly believe that the here presented findings will contribute to a more rational design of biological and clinical studies, hopefully enabling identification of adequate prognosticators in OPLs and molecular pathways to be addressed for reduction of the risk of malignant transformation.

## Supplementary Materials

The following are available online at https://www.mdpi.com/2073-4409/9/8/1828/s1, Figure S1: Gene Set Enrichment Analysis (GSEA), Figure S2: Tumor microenvironment composition (xCell), Table S1: Prognostic signatures
