# Supplementary figures and images for: Gene Expression Clustering and Selected Head and Neck Cancer Gene Signatures Highlight Risk Probability Differences in Oral Premalignant Lesions
Source: Cells. 2020 Aug 3;9(8):1828. doi: 10.3390/cells9081828 (PMC7466020; doi:10.3390/cells9081828)

Strata

CI1 CI3 CI5  
CI2 CI4 CI6

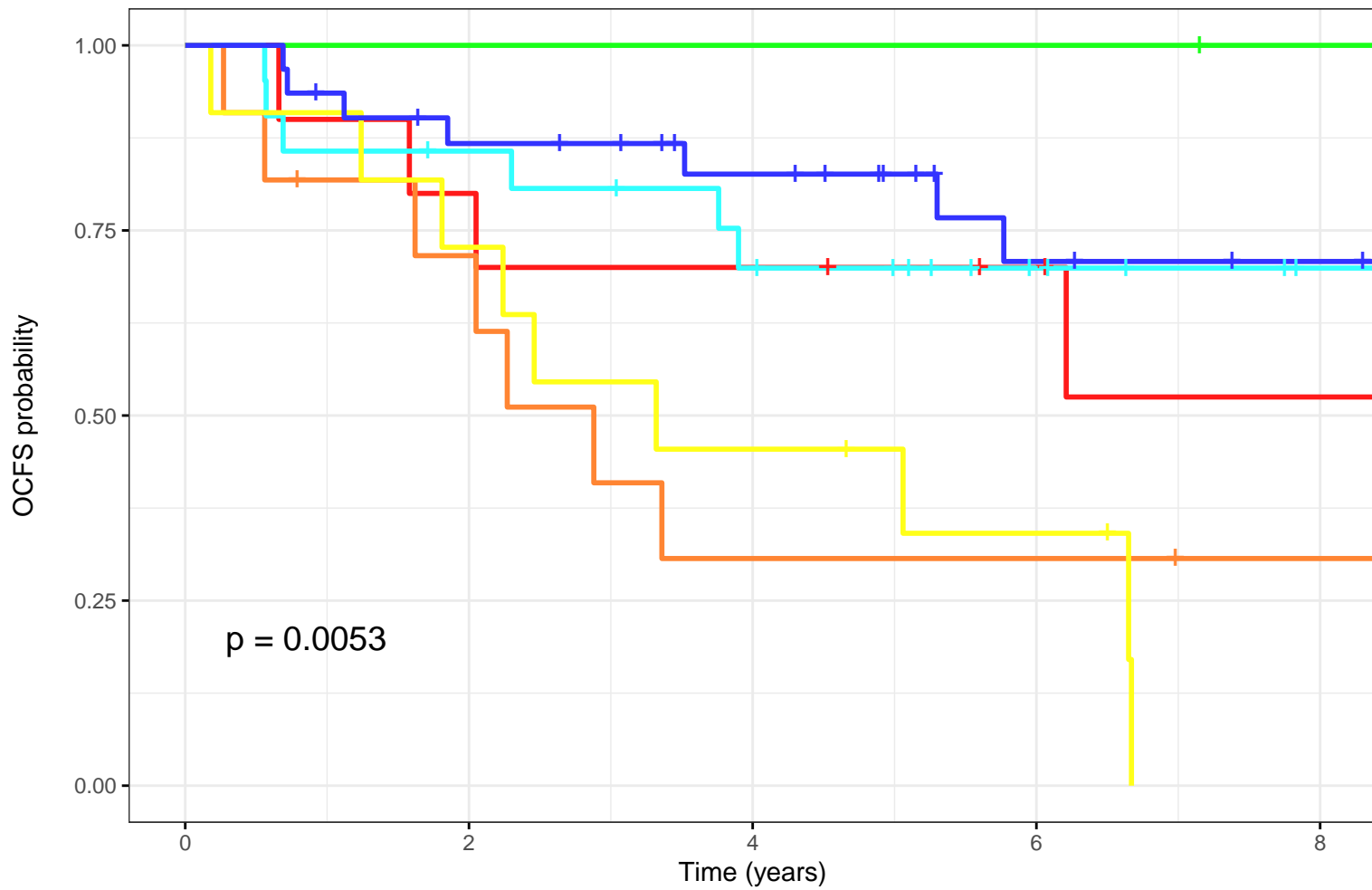

Number at risk

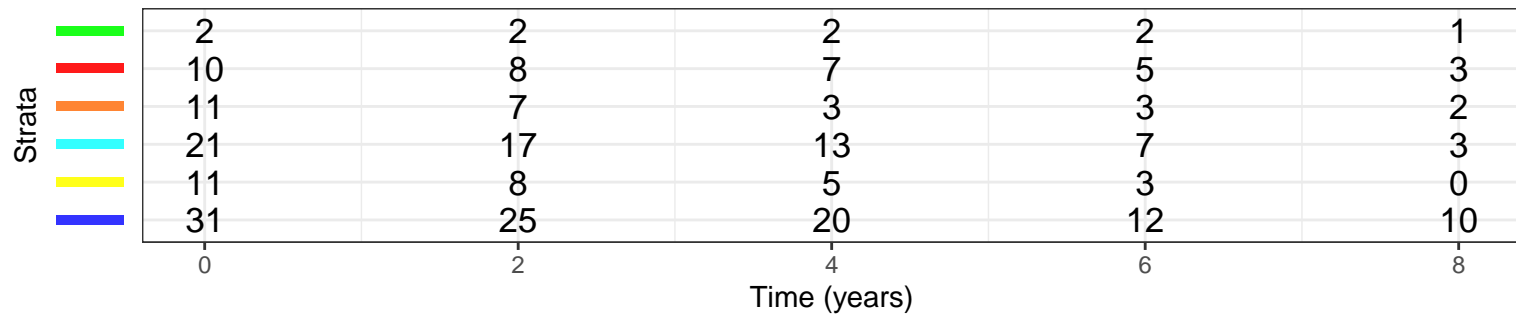

Supplement: Supplementary file 1 [file cells-09-01828-s001.zip › Figures/Figure1.pdf]

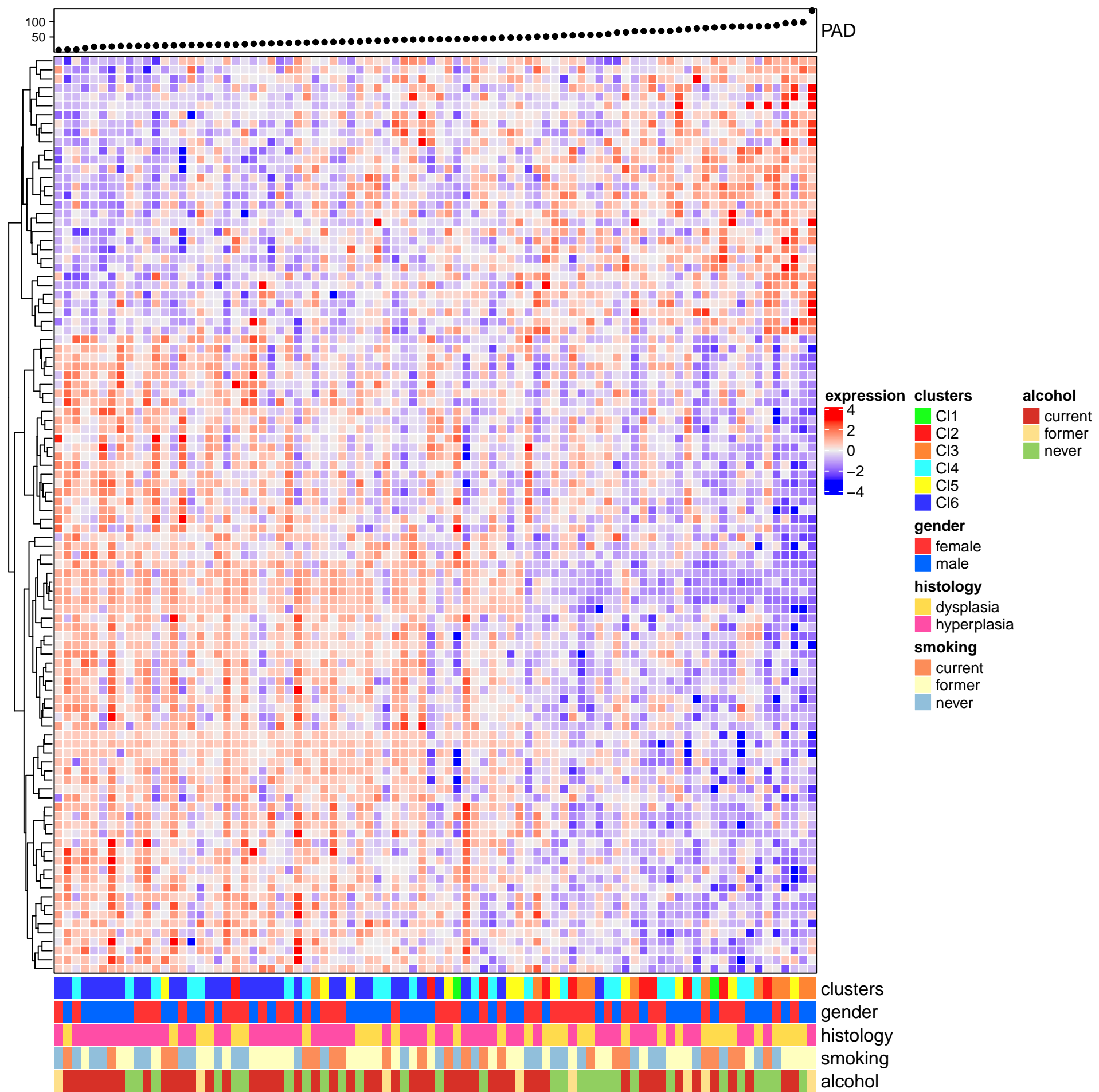

Supplement: Supplementary file 1 [file cells-09-01828-s001.zip › Figures/Figure2.pdf]

**A**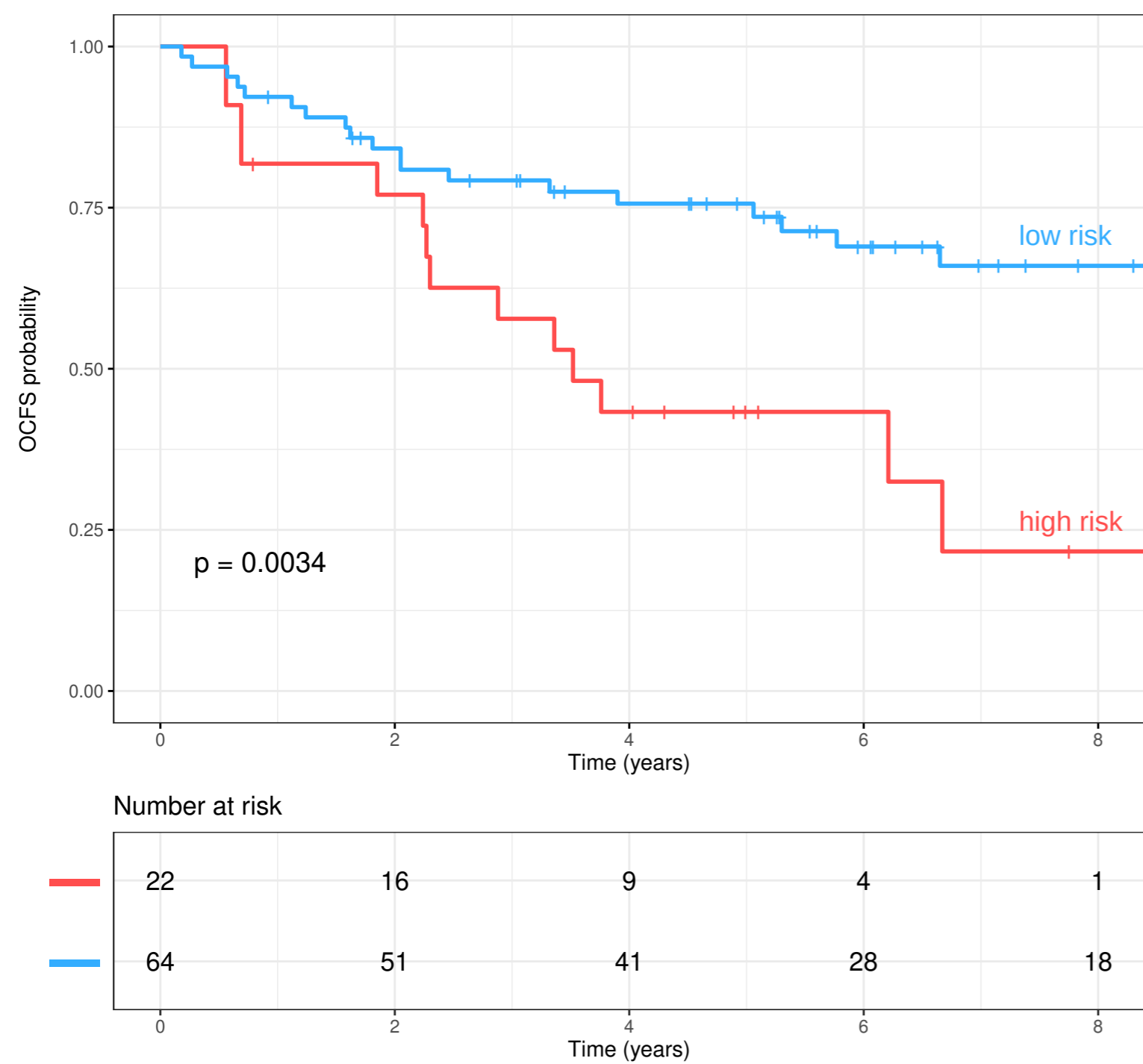**B**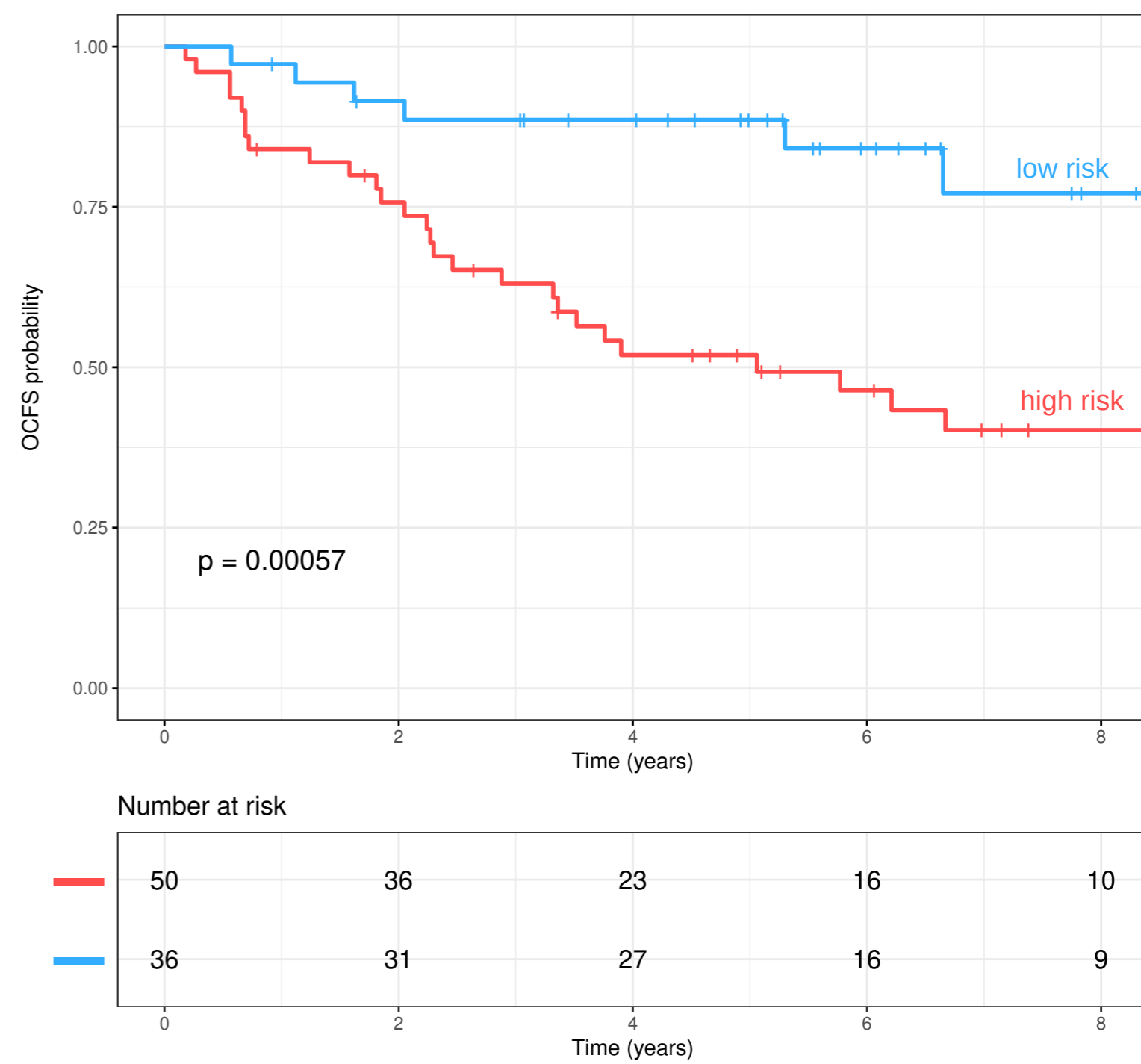**C**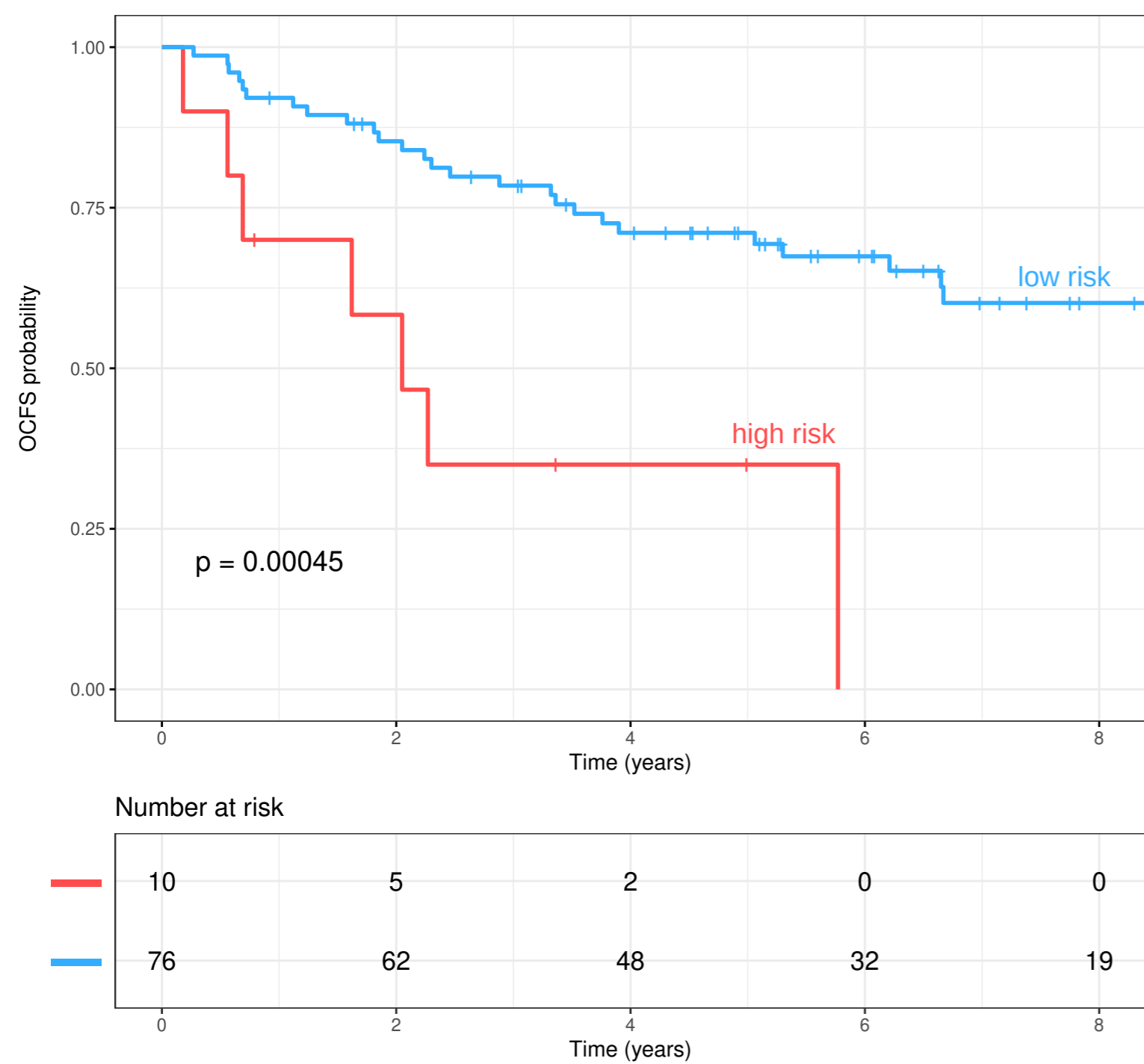**D**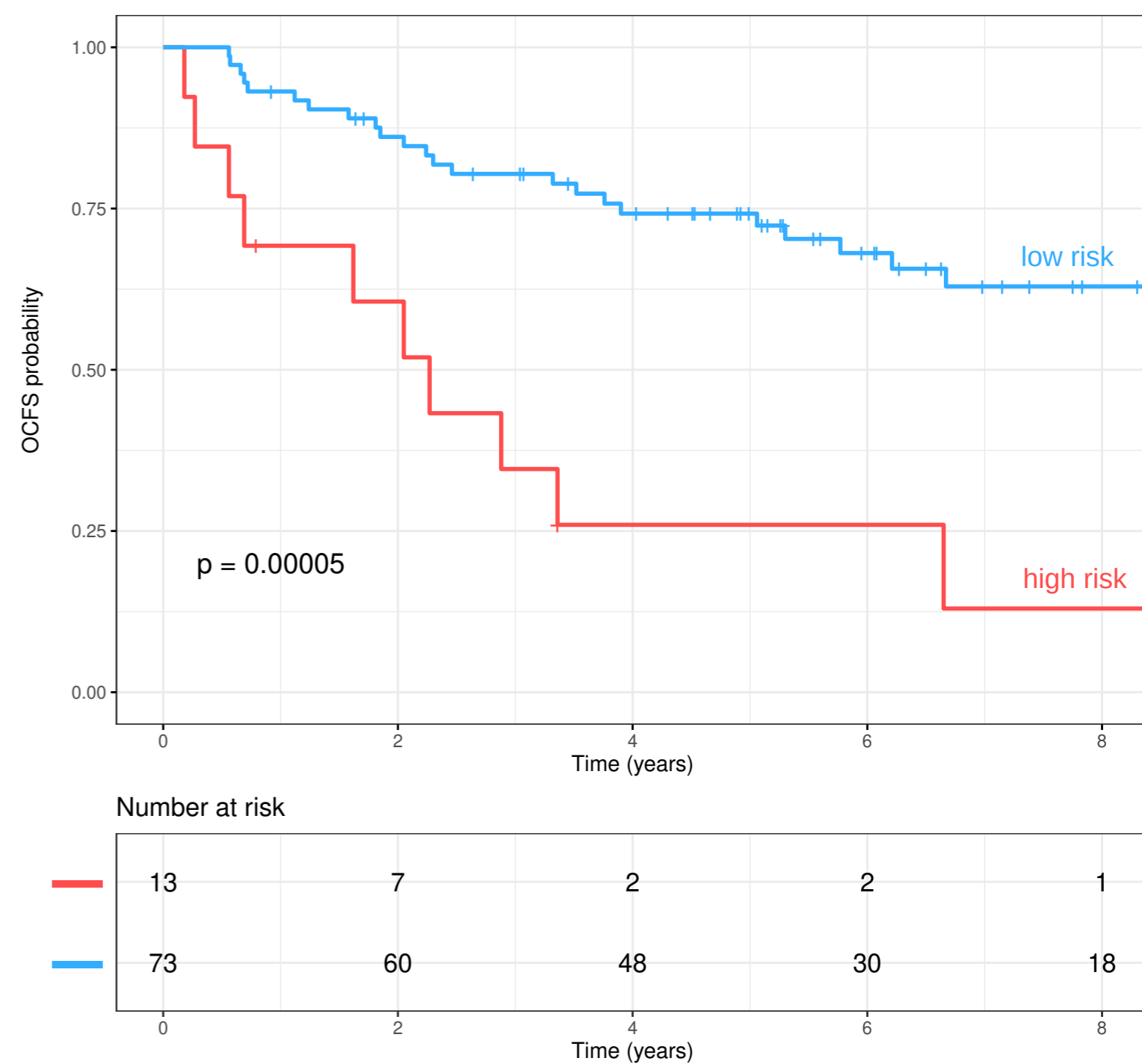

Supplement: Supplementary file 1 [file cells-09-01828-s001.zip › Figures/Figure3.pdf]

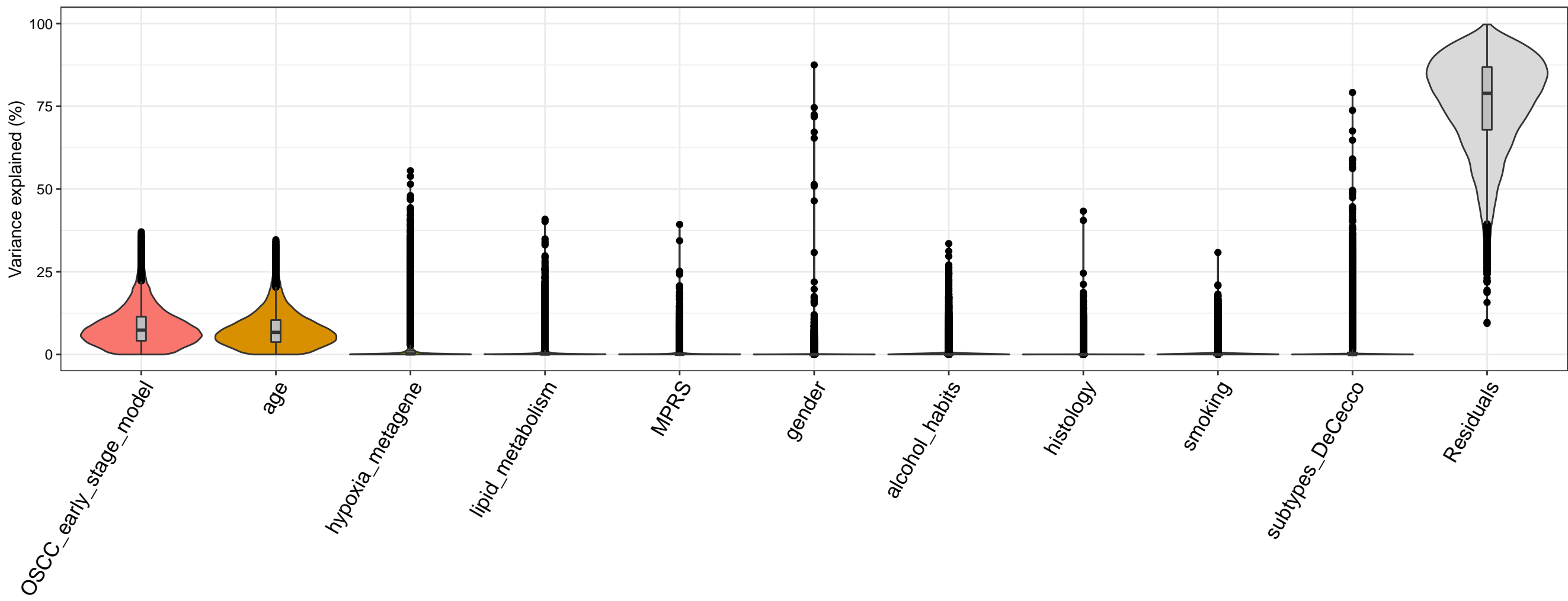

Supplement: Supplementary file 1 [file cells-09-01828-s001.zip › Figures/Figure4.pdf]
